# Comparison of the Characteristics and Risk Factors of Carotid Atherosclerosis in High Stroke Risk Populations Between Urban and Rural Areas in North China

**DOI:** 10.3389/fneur.2020.554778

**Published:** 2020-11-09

**Authors:** Jin Zhang, Hui Sang, Xin Zhang, Yalan Fang, Xiaoyuan Niu, Tingting Liu, Weidong Liu, Jian Li

**Affiliations:** ^1^Department of Neurology, The First Hospital of Shanxi Medical University, Taiyuan, China; ^2^Department of Neurology, Taiyuan Central Hospital, Taiyuan, China; ^3^Department of Neurology, Coal Group General Hospital, Datong, China; ^4^Neurosurgical Department, Liaocheng People's Hospital, Liaocheng, China; ^5^Department of Neurology, Affiliated Hospital of Weifang Medical University, Weifang, China

**Keywords:** carotid atherosclerosis, northern China, high stroke risk, urban, rural

## Abstract

**Objective:** To study the characteristics and risk factors of carotid atherosclerosis in populations at high risk of stroke in urban and rural areas of North China.

**Methods:** A cross-sectional study was conducted to investigate high stroke risk populations in representative urban and rural areas sampled from 12 regions of China. A pre-designed questionnaire, ultrasound, and laboratory examinations were performed to evaluate risk factors.

**Results:** A total of 30,175 patients were included in the study. The overall prevalence of carotid atherosclerosis was 54.53%, among which intimal thickening and plaque were 39.22 and 41.25%, respectively. The prevalence of carotid atherosclerosis in the urban group was higher than in the rural group. Multivariate logistic regression analysis revealed that male gender, age, smoking, hypertension, dyslipidemia, stroke, atrial fibrillation, systolic blood pressure, and levels of fasting blood glucose, total cholesterol, and low-density lipoprotein cholesterol were the common independent risk factors for carotid atherosclerosis in both groups. Higher education, high salt consumption, passive smoking, family history of stroke, and transient ischemic attack were unique independent risk factors, and high-density lipoprotein cholesterol was a protective factor for carotid atherosclerosis in the urban population.

**Conclusion:** This study suggests that risk factors for carotid atherosclerosis differ between urban and rural populations in North China.

## Introduction

Cardiovascular disease is the leading cause of death in the world. The mortality rate associated with cardiovascular disease is the highest among non-communicable diseases and is still gradually rising. Stroke accounts for a large proportion of cardiovascular disease (1, 2). In China, the overall prevalence of stroke is 2.06%, and the prevalence rate is higher in rural areas compared to urban areas (3). Notably, the incidence of stroke is increasing by 8% (4). Atherosclerosis is an important pathogenesis underlying stroke and can slowly develop for decades before clinical manifestations and endpoint events occur (5). Due to the superficial position of the carotid artery, ultrasound is often used to directly detect carotid atherosclerosis (CAS), and CAS has been shown to be a strong predictor of stroke events in many studies (6). The majority of recent studies on carotid atherosclerosis in China were regional and community-based, which reported that the prevalence of CAS ranges from 22.3 to 72.5% (7–9). However, there are limited studies focusing on the characteristics and differences of CAS risk factors between urban and rural populations. Varied prevalence of CAS among different areas may be due to distinct exposure to risk factors (10). In this study, we compared the characteristics and risk factors of CAS in high stroke risk populations between urban and rural areas of North China to provide evidence for the development of precise preventive measures to reduce the incidence of CAS and stroke.

## Materials and Methods

### Study Population

This was a cross-sectional study conducted from August 2016 to May 2017. The study population was obtained from the 2016 Stroke Screening and Prevention Program of the National Health and Family Planning Commission of China (10). Individuals were sampled from northern China, including Beijing, Hebei, Shanxi, Henan, Shaanxi, Gansu, Shandong, Jiangsu, Liaoning, Jilin, Heilongjiang, and Inner Mongolia. According to the geographical location and the criteria of division in China, the regions were divided into urban and rural areas (10). The regional distribution of sampling was reasonable and representative. Regional population files were complete, and transportation in these areas was convenient (10). Residents who had lived in these areas for more than 6 months and were over 40 years of age were included. Considering a village or a community as a unit, we performed cluster random sampling of eligible populations at screening sites; 2,000 people from each site were sampled. To screen populations at high risk of stroke, basic demographic information and risk factors for stroke were collected, and physical, ultrasound and laboratory examinations were performed (4). After excluding cases with incomplete data, we included a total of 30,175 individuals, including 14,461 cases in urban areas and 15,714 cases in rural areas. The overall response rate of the population was ≥85%, and mean age of the population was 62.54 ± 9.77 years. The research protocol was approved by the Ethics Clerk Association of the First Hospital of Shanxi Medical University. Written informed consent was obtained from all subjects.

### Research Methods and Standards

Using a standardized epidemiological survey questionnaire, this study was conducted through face-to-face manner by trained research staff. A multi-level quality control team was set up to check the contents of the investigation, correct the error information promptly, and report the data in real time.

The diagnostic criteria of risk factors were the following (10): hypertension, defined as a history of high blood pressure (≥140/90 mmHg) or taking antihypertensive medications; atrial fibrillation (AF), diagnosed by electrocardiogram during physical exam; diabetes mellitus, defined as a previous diagnosis, receiving treatment of insulin or oral hypoglycemic medications, fasting blood glucose (FBG) ≥ 7.0 mmol/L, or glycosylated hemoglobin ≥ 6.5%; dyslipidemia, defined as taking antilipidemic medications or one or more abnormalities in laboratory examinations, including total cholesterol (TC) ≥ 6.22 mmol/L (240 mg/dl), total triglyceride (TG) ≥ 2.26 mmol/L (200 mg/dl), low-density lipoprotein cholesterol (LDL-C) ≥ 4.14 mmol/L (160 mg/dl), or high-density lipoprotein cholesterol (HDL-C) < 1.04 mmol/L (40 mg/dl); smoking, defined as current practice of smoking; passive smoking refers to non-smokers who have been exposed to smoke for at least 15 min a day for more than 1 day a week; lack of exercise, defined as physical exercise <3 times a week with <30 min each time (industrial and agricultural labor was considered exercise); overweight, defined as body mass index (BMI) ≥ 26 kg/m^2^; obesity, defined as BMI ≥ 28 kg/m^2^; and family history of stroke. Patients with ≥3 risk factors or previous history of stroke or transient ischemic attack (TIA) were defined as high stroke risk populations.

Cervical vascular ultrasound examination was performed with standardized technology (11). The subjects were placed in a supine position with the head on the opposite side of the examination site. Extracranial carotid arteries, which include the common carotid artery (CCA), bifurcation, and internal and external carotid arteries on both sides, were screened for arterial intima-media thickness (IMT), plaques, and stenosis (10). IMT ≥ 1.0 mm was considered as thickened; IMT ≥ 1.5 mm was defined as plaque. The degree of carotid artery stenosis was divided into non-stenosis, 1–49% (mild), 50–69% (moderate), 70–99% (severe), and occlusive (10).

### Statistical Analysis

Statistical analysis was performed with the IBM SPSS Statistics software (version 24.0). Quantitative data were expressed as mean ± standard deviation (SD). If normal distribution was confirmed, independent sample *t* tests were used; otherwise, two independent sample non-parametric tests were performed. The qualitative data were expressed as frequencies and percentages, and the chi-square test was used for comparisons between groups. The multivariate logistic regression analysis was constructed to analyze the risk factors for carotid atherosclerosis in different groups. *p* < 0.05 in the two-sided tests was considered significant.

## Results

### Characteristics of the Population at High Risk of Stroke

The demographic and clinical characteristics of the high stroke risk populations are listed in Table 1. Among the participants, 20,785 patients had ≥3 stroke risk factors, and 6,032 and 3,358 patients had prior stroke and TIA, respectively. The prevalence of risk factors in this population-based study was ranked from high to low as follows: hypertension (73.41%), high salt consumption (≥6 g/day, 58.11%), lack of exercise (54.62%), overweight or obesity (54.02%), dyslipidemia (45.75%), family history of stroke (36.09%), smoking (30.01%), diabetes (25.56%), drinking (21.49%), history of previous stroke (19.99%), history of previous TIA (11.13%), passive smoking (5.36%), and AF (6.37%).

**Table 1:** Characteristics of the population at high risk of stroke.

**Risk factors**		**Total**	**Urban**	**Rural**	***p***
		***n* = 30,175**	***n* = 14,461**	***n* = 15,714**	
Age		62.5 ± 9.8	62.8 ± 9.9	62.3 ± 9.7	<0.001
Sex	Male	13,903 (46.1%)	6,645 (45.9%)	7,258 (46.2%)	0.680
Smoking	Yes	9,055 (30.0%)	3,905 (27.0%)	5,150 (32.8%)	<0.001
	Passive smoking	1,617 (5.4%)	670 (4.6%)	947 (6.0%)	
Alcohol intake		6,485 (21.5%)	3,091 (21.4%)	3,394 (21.6%)	<0.001
High salt consumption		16,965 (58.1%)	7,518 (54.2%)	9,447 (61.7%)	<0.001
Average salt intake (g/day)		7.1 ± 5.7	6.9 ± 7.1	7.2 ± 3.9	0.001
Lack of exercise		16,481 (54.6%)	8,534 (59.0%)	7,947 (50.6%)	<0.001
Family history of stroke		10,889 (36.1%)	5,048 (34.9%)	5,841 (37.2%)	<0.001
History of previous TIA		3,358 (11.1%)	1,596 (11.0%)	1,762 (11.2%)	0.627
History of previous stroke		6,032 (20.0%)	2,502 (17.3%)	3,530 (22.5%)	<0.001
Hypertension		22,150 (73.4%)	10,333 (71.5%)	11,817 (75.2%)	<0.001
Treatment of hypertension			7,553 (73.1%)	7,973 (67.5%)	<0.001
Control of hypertension			6,303 (61.0%)	6,192 (52.4%)	<0.001
Diabetes		8,015 (26.6%)	4,433 (30.6%)	3,582 (22.8%)	<0.001
Treatment of diabetes			2,868 (64.7%)	2,027 (56.6%)	<0.001
Control of diabetes			2,629 (59.3%)	1,967 (54.9%)	<0.001
Dyslipidemia		13,805 (45.8%)	7,461 (51.6%)	6,344 (40.4%)	<0.001
AF		1,922 (6.4%)	1,045 (7.2%)	877 (5.6%)	<0.001
Overweight or obesity		16,300 (54.0%)	7,910 (54.7%)	8,390 (53.4%)	0.023
SBP (mmHg)		139.5 ± 18.3	137.9 ± 17.2	140.9 ± 19.0	<0.001
DBP (mmHg)		84.9 ± 10.8	83.7 ± 10.5	86.0 ± 10.9	<0.001
FBG (mmol/L)		6.0 ± 1.8	6.1 ± 1.8	5.9 ± 1.8	<0.001

*TIA, transient ischemic attack; AF, atrial fibrillation; SBP, systolic blood pressure; DBP, diastolic blood pressure; FBG, fasting blood glucose*.

Age, FBG, levels of education and income, and the percentages of diabetes, dyslipidemia, atrial fibrillation, lack of exercise, and overweight or obesity in the urban group were significantly higher than in the rural group (Table 1, *p* < 0.05 or *p* < 0.01). Systolic (SBP) and diastolic (DBP) blood pressure and the percentages of smoking, drinking, high salt consumption, family history of stroke, previous stroke, and hypertension in the rural group were higher than in the urban group (Table 1, *p* < 0.05 or *p* < 0.01).

### Prevalence of CAS

**Figure 1:**
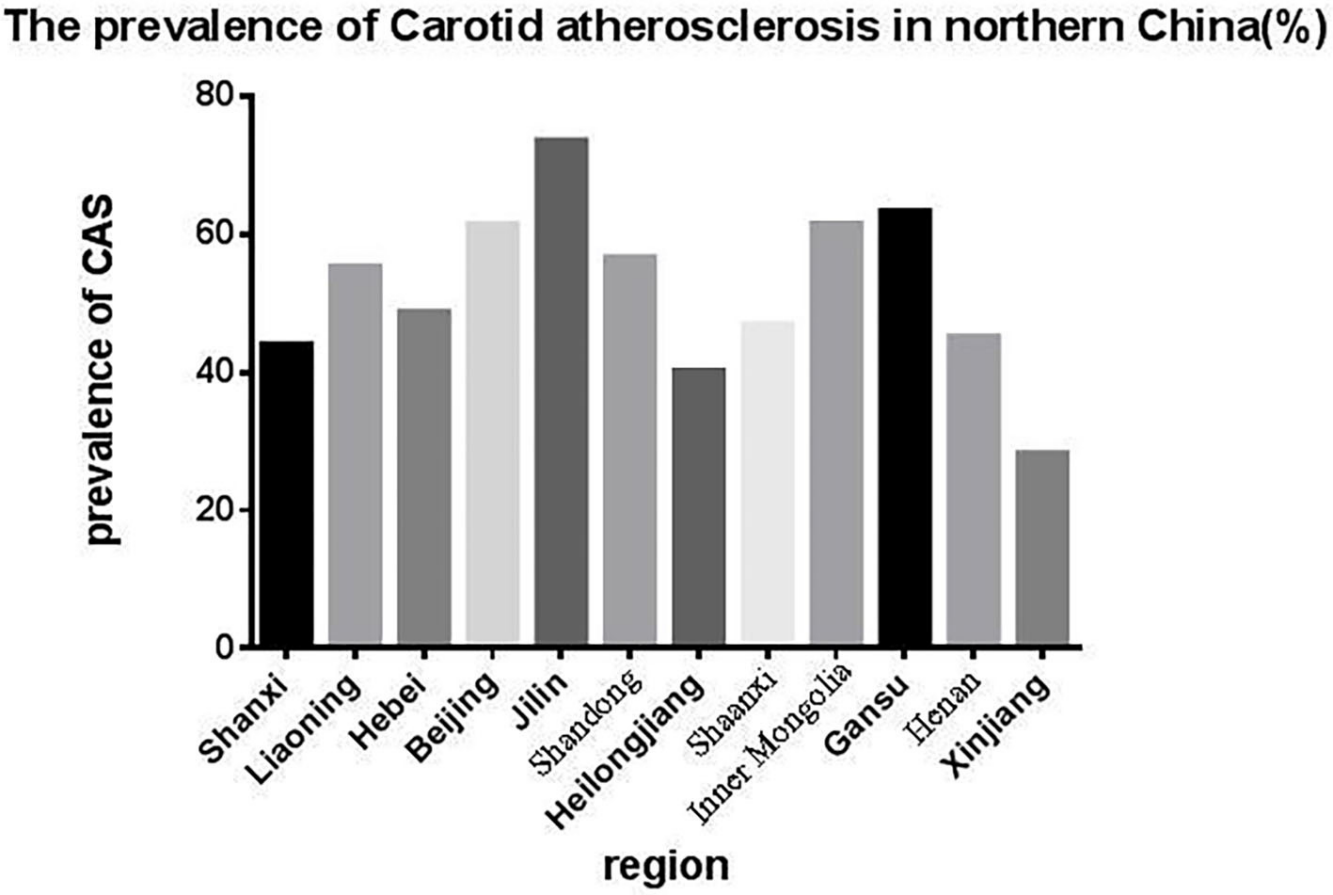
The prevalence of CAS in northern China, which was highest in Jilin and lowest in Xinjiang.

The overall prevalence of CAS in the population was 54.53%, with a higher prevalence in the urban group (56.47%) than the rural group (52.74%) (Table 2, *p* < 0.01). More specifically, Jilin (73.5%) had the highest prevalence of CAS, followed by Gansu (63.3%), Inner Mongolia (61.5%), Beijing (61.4%), Shandong (56.6%), Liaoning (55.3%), Hebei (48.7%), Shaanxi (46.9%), Henan (45.1%), Shanxi (44.0%), and Heilongjiang (40.2%); Xinjiang (28.8%) had the lowest prevalence (Figure 1). We also compared the prevalence of CAS in rural and urban area.

**Table 2:** Prevalence and characteristics of CAS in urban and rural populations.

		**Total**	**Urban**	**Rural**	***p* values**
		**(*N* = 30,175)**	**(*N* = 14461)**	**(*N* = 15714)**	
CAS		16,454 (54.5%)	8,166 (56.5%)	8,288 (52.7%)	<0.001
IMT thickening		11,835 (39.2%)	6,141 (42.5%)	5,694 (36.2%)	<0.001
Plaque formation		12,448 (41.3%)	6,330 (43.8%)	6,118 (38.9%)	<0.001
Stenosis		1,221 (4.1%)	667 (4.6%)	554 (3.5%)	<0.001
Stenosis rate	1–49%	1,051 (3.5%)	590 (4.1%)	461 (2.9%)	<0.001
	50–69%	75 (0.3%)	30 (0.2%)	45 (0.3%)	
	70–99% or	95 (0.3%)	47 (0.3%)	48 (0.3%)	
	occlusive				

*CAS, carotid atherosclerosis; IMT, intima-media thickness*.

### Risk Factors for CAS in the Urban Group

The prevalence of CAS among individuals with different characteristics in the urban group are compared in Table 3. The prevalence of CAS was higher in males and individuals with higher education and income levels, smoking, alcohol intake, lack of exercise, family history of stroke, hypertension, dyslipidemia, diabetes, overweight or obesity, over 80 years of age, daily intake of 6 g or more salt, history of previous TIA, history of previous stroke, and AF (Table 3, *p* < 0.05 or *p* < 0.01). Compared with normal individuals in the urban population, patients with CAS had higher levels of SBP, FBG, TC, LDL, and HDL (all *p* < 0.01) but had similar levels of DBP and TG (Table 4).

**Table 3:** Prevalence of CAS among urban individuals with different characteristics.

		**CAS group**	**Rate%**	***p* values**
Gender	Male	4,003	60.2	< 0.001
	Female	4,163	53.3	
Smoking	No	5,376	54.4	< 0.001
	Yes	2,393	61.3	
	Passive smoking	397	59.3	
Alcohol intake	No	6,228	54.8	< 0.001
	Yes	1,938	62.7	
Exercise habit	Often	3,541	59.7	< 0.001
	Lack	4,625	54.2	
Family history of stroke	Yes	2,990	59.2	< 0.001
	No	5,176	55.0	
Hypertension	Yes	6,031	58.4	< 0.001
	No	2,135	51.7	
Dyslipidemia	Yes	4,576	61.3	< 0.001
	No	3,590	51.3	
Diabetes	Yes	2,581	58.2	0.005
	No	5,585	55.7	
Overweight or obesity	Yes	4,302	54.4	< 0.001
	No	3,864	59.0	
Age	40–59 years	2,236	42.2	< 0.001
	60–79 years	5,477	64.7	
	Over 80 years	453	65.2	
Average salt intake (g/day)	≤5.99	3,447	54.2	< 0.001
	≥6	4,351	57.9	
History of previous TIA	Yes	984	61.7	< 0.001
	No	7,182	55.8	
History of previous stroke	Yes	1,726	69.0	< 0.001
	No	6,440	53.9	
AF	Yes	645	61.7	< 0.001
	No	7,521	56.1	

*CAS, carotid atherosclerosis; TIA, transient ischemic attack; AF, atrial fibrillation*.

**Table 4:** Comparison of parameters between normal and CAS individuals in the urban population.

	**Normal**	**CAS**	***p***
SBP (mmHg)	134 (125–142)	138 (130–150)	<0.001
DBP (mmHg)	82 (78–90)	82 (78–90)	0.210
FBG (mmol/L)	5.5 (5–6.1)	5.7 (5.2–6.7)	<0.001
TG (mmol/L)	1.6 (1.2–2.2)	1.6 (1.1–2.2)	0.213
TC (mmol/L)	4.9 (4.0–5.4)	5.0 (4.3–5.8)	<0.001
LDL (mmol/L)	2.7 (2.1–3.3)	2.9 (2.2–3.6)	<0.001
HDL (mmol/L)	1.3 (1.1–1.7)	1.3 (1.1–1.6)	<0.001

*CAS, carotid atherosclerosis; SBP, systolic blood pressure; DBP, diastolic blood pressure; FBG, fasting blood glucose; TG, total triglyceride; TC, total cholesterol; LDL, low-density lipoprotein cholesterol; HDL, high-density lipoprotein cholesterol*.

Multivariate logistic regression analysis showed that age, male gender, smoking, passive smoking, hypertension, dyslipidemia, high salt consumption, family history of stroke, history of previous TIA and stroke, AF, and high level of SBP, TC, LDL, and FBG were independent risk factors for CAS in the urban population, and HDL was a protective factor (Table 5).

**Table 5:** Regression analysis of CAS risk factors in the urban population.

		***p* values**	**OR (95% CI for OR)**
SBP		<0.001	1.011 (1.008–1.013)
FBG		<0.001	1.140 (1.112–1.168)
TC		<0.001	1.100 (1.066–1.134)
LDL		0.002	1.049 (1.018–1.080)
HDL		<0.001	0.830 (0.783–0.880)
High salt consumption		0.001	1.128 (1.049–1.213)
Age	60–79 years	<0.001	2.602 (2.405–2.814)
	Over 80 years	<0.001	3.051 (2.558–3.639)
Smoking	Yes	<0.001	1.274 (1.145–1.418)
	Passive smoking	0.005	1.286 (1.080–1.530)
Family history of stroke	Yes	<0.001	1.366 (1.262–1.479)
Hypertension	Yes	<0.001	1.196 (1.096–1.305)
Dyslipidemia	Yes	<0.001	1.502 (1.393–1.619)
Sex	Male	<0.001	1.178 (1.076–1.289)
TIA	Yes	<0.001	1.581 (1.401–1.784)
Stroke	Yes	<0.001	1.855 (1.672–2.056)
AF	Yes	0.003	1.355 (1.110–1.654)

*SBP, systolic blood pressure; FBG, fasting blood glucose; TC, total cholesterol; LDL, low-density lipoprotein cholesterol; HDL, high-density lipoprotein cholesterol; TIA, transient ischemic attack; AF, atrial fibrillation*.

### Risk Factors for CAS in the Rural Population

The prevalence of CAS among individuals is compared with different characteristics in the rural population in Table 6. Males and individuals with smoking, alcohol intake, hypertension, dyslipidemia, overweight or obesity, over 80 years of age, history of previous TIA and stroke, and AF had higher prevalence rates of CAS in the rural population (Table 6, *p* < 0.01)., Compared with normal individuals in the rural population, patients with CAS had higher levels of SBP, DBP, FBG, TC, and LDL (all *p* < 0.01) but had similar levels of TG and HDL (Table 7).

**Table 6:** Prevalence of CAS among rural individuals with different characteristics.

		**CAS group**	**Rate%**	***p***
Gender	Male	4,000	55.1	< 0.001
	Female	4,288	50.7	
Smoking	No	4,917	51.1	< 0.001
	Yes	2,879	55.9	
	Passive smoking	492	52.0	
Alcohol intake	No	6,382	51.8	< 0.001
	Yes	1,906	56.2	
Exercise habit	Often	4,056	52.2	0.195
	Lack	4,232	53.3	
Family history of stroke	Yes	3,087	52.9	0.835
	No	5,201	52.7	
Hypertension	Yes	6,420	54.3	< 0.001
	No	1,868	47.9	
Dyslipidemia	Yes	3,501	55.2	< 0.001
	No	4,787	51.1	
Diabetes	Yes	1,932	53.9	0.103
	No	6,356	52.4	
Overweight or obesity	Yes	4,315	51.4	< 0.001
	No	3,973	54.3	
Age	40–59 years	2,356	39.1	< 0.001
	60–79 years	5,508	60.9	
	Over 80 years	424	65.5	
Average salt intake (g/day)	≤5.99	3,073	52.4	0.767
	≥6	4,924	52.1	
History of previous TIA	Yes	826	46.9	< 0.001
	No	7,462	53.5	
History of previous stroke	Yes	2,150	60.9	< 0.001
	No	6,138	50.4	
AF	Yes	574	65.5	< 0.001
	No	7,714	52.0	

*CAS, carotid atherosclerosis; TIA, transient ischemic attack; AF, atrial fibrillation*.

**Table 7:** Comparison of parameters between normal and CAS individuals in the rural population.

	**Normal**	**CAS**	***p***
SBP (mmHg)	138 (128–148)	140 (130–155)	<0.001
DBP (mmHg)	85 (80–90)	86 (80–92)	<0.001
FBG (mmol/L)	5.4 (4.9–6.1)	5.6 (5.0–6.4)	<0.001
TG (mmol/L)	1.5 (1.2–2.1)	1.5 (1.1–2.2)	0.062
TC (mmol/L)	4.8 (4.0–5.5)	5 (4.3–5.8)	<0.001
LDL (mmol/L)	2.6 (2.0–3.2)	2.8 (2.2–3.4)	<0.001
HDL (mmol/L)	1.4 (1.1–1.7)	1.3 (1.1–1.6)	0.085

*CAS, carotid atherosclerosis; SBP, systolic blood pressure; DBP, diastolic blood pressure; FBG, fasting blood glucose; TG, total triglyceride; TC, total cholesterol; LDL, low-density lipoprotein cholesterol; HDL, high-density lipoprotein cholesterol*.

Multivariate logistic regression analysis showed that age, male gender, smoking, hypertension, dyslipidemia, history of previous stroke, AF, and high levels of SBP, TC, LDL, and FBG were independent risk factors for CAS in the rural population (Table 8).

**Table 8:** Regression analysis of CAS risk factors in the rural population.

		**B**	***p***	**OR (95% CI for OR)**
SBP		0.008	<0.001	1.008 (1.006–1.010)
FBG		0.086	<0.001	1.090 (1.069–1.112)
TC		0.132	<0.001	1.141 (1.108–1.175)
LDL		0.031	0.027	1.031 (1.003–1.060)
Age	60–79 years	0.832	<0.001	2.297 (2.140–2.465)
	Over 80 years	1.072	<0.001	2.922 (2.451–3.483)
Smoking	Yes	0.245	<0.001	1.277 (1.166–1.398)
Hypertension	Yes	0.122	<0.001	1.130 (1.040–1.227)
Dyslipidemia	Yes	0.160	<0.001	1.174 (1.095–1.258)
Sex	Male	0.139	0.001	1.149 (1.057–1.249)
Stroke	Yes	0.389	<0.001	1.476 (1.357–1.605)
AF	Yes	0.599	<0.001	1.820 (1.564–2.117)

*SBP, systolic blood pressure; FBG, fasting blood glucose; TC, total cholesterol; LDL, low-density lipoprotein cholesterol; AF, atrial fibrillation*.

Men, age, smoking, hypertension, dyslipidemia, stroke, AF, and high level of SBP, FBG, TC, and LDL were common risk factors for CAS in both urban and rural groups. High salt consumption, high levels of education, passive smoking, family history of stroke, and TIA were specific risk factors for the urban group, and HDL was a protective factor for the urban group.

## Discussion

A number of population-based studies have shown that the incidence of ischemic stroke has been increasing rapidly (12, 13). With the growth of the aging population, more time and money are being spent on the functional health loss caused by stroke, which has increased social and economic burdens (14). CAS is the main risk factor for ischemic stroke; CAS becomes clinically overt after a long-term subclinical process (15). In addition to CAS, hypertension is identified as a key risk factor for ischemic stroke (16). Several studies have shown that carotid IMT and plaque formation are strong predictors of future stroke events (6, 17). Furthermore, hypertension, smoking, overweight or obesity, dyslipidemia, and diabetes are all risk factors for both stroke and CAS (8, 15, 18). Differences in the prevalence of risk factors may determine the prevalence of CAS in certain populations (2).

This study included most cities in northern China. The overall prevalence of CAS was 54.53% in the high stroke risk population, with 57% in urban areas and 54.3% in rural areas. Results also showed that Jilin had the highest rate of CAS, while Xinjiang had the lowest one, which may be due to the differences in sample areas and populations. As for the prevalence of carotid plaques, IMT, and stenosis, it varies widely (14, 19). Our data show that the prevalence of IMT, plaque, and stenosis were 39.22, 41.25, and 4.05%, respectively. The common risks for CAS in both urban and rural groups reported in our study were consistent with numerous previous studies. For example, risk factors for carotid plaque and IMT include age, sex, smoking, diabetes, hypertension, high FBG and LDL levels, decreased HDL-C levels, and history of stroke (19, 20).

Many risk factors have been reported as independent predictors of CAS (21). Age is an independent risk factor for CAS. According to the regression analysis, the prevalence rate of CAS remarkably increased with age, which was consistent with previous studies (22). In the urban group, the CAS risk of individuals >80 years of age and 60–79 years of age were 3.051 and 2.602 times higher compared to the 40–59 age group, respectively, and 2.922 and 2.297 times higher compared to the rural group, respectively. Gender was also closely related to the occurrence of CAS. Compared to women, men were at a higher risk of CAS, which may be due to estrogen levels (23). In addition, smoking is one of the risk factors of CAS, including active smoking and passive smoking. Previous studies indicated that smoking was an independent risk factor for CAS, and active smoking plays an important role in the progression of CAS, with a clear dose-response relationship (7, 8, 24). China is the largest tobacco producer and consumer in the world (25). The prevalence of active smoking among Chinese adults has dropped by 19.5% (9) with the popularization of health concepts. However, the prevalence of passive smoking has exceeded 50%, which mostly affects women and children (9). Children with smoking parents have an increased risk of CAS plaques in adulthood (26). In this study, the proportion of passive smoking was higher in the rural population compared to the urban population, while the prevalence of CAS in passive smokers was higher in the urban group compared to the rural group. Based on the multivariate analysis, passive smoking was a unique risk factor for CAS in urban residents. A possible reason may be that people in urban areas live in a more closed environment where smoke lasts longer due to poor air circulation. Luckily, passive smoking is a preventable factor, and with increased awareness of tobacco hazards and strengthened formulation and implementation of public health policies, the rates of smoking and passive smoking have gradually declined (25).

The prevalence of hypertension and dyslipidemia varies in rural and urban areas. Hypertension and elevated SBP were common CAS risk factors, as reported in previous studies (7, 18, 27). Elevated blood pressure, especially SBP, alters vascular wall tension and shear stress, which can cause damage to vascular endothelial cells and promote atherosclerosis (18). In this study, the prevalence of hypertension in the study population was 73.41%, with the rate being higher in the rural group compared to the urban group (75.20 vs. 71.45%), however, the treatment and control rates of hypertension were higher in the urban group. Similarly, one previous report also indicated that people in townships have higher prevalence rate of hypertension than people in urban areas, but the overall rates of awareness, treatment, and control were lower than in urban areas, which may be due to lower education or lower annual income (28). Thus, effective blood pressure management is an important measure to reduce the burden of disease. Meanwhile, for the rural population, it is also necessary to increase investment in education and to popularize the knowledge of disease and medical resources. As for dyslipidemia, it has higher prevalence in the urban population compared to the rural population. Among individuals with dyslipidemia, the prevalence of CAS was higher in urban areas compared to the rural areas. High LDL-C and TC levels were common risk factors for CAS in both groups, and HDL was a protective factor in the urban population. This phenomenon may be related to the progress of social mechanization and the decline in physical activity (3). In recent years, the urban population has tended to adopt a more Westernized diet, which contains more fat, protein, salt, and sugar than traditional Chinese foods (29). Meanwhile, the dietary structure of the rural population also has changed, resulting in increased prevalence of obesity and dyslipidemia (10). In addition, the sedentary work style and lack of exercise gradually increase the incidence of dyslipidemia and obesity.

In this study, we identified various risk factors for carotid atherosclerosis between urban and rural populations in North China. It is the first time to explore the population for CAS in rural and urban areas. However, some limitations need to be noticed. First, this is a cross-sectional study that cannot predict the development of CAS and stroke. Prospective cohort studies are needed. Secondly, this study only analyzed traditional risk factors. Third, the study has selection bias, especially among the healthy controls.

## Conclusion

This study found that traditional risk factors for CAS, such as male gender, age, smoking, hypertension, and dyslipidemia were common in urban and rural groups. Additionally, high salt consumption and passive smoking were significant risk factors in the urban population. A variety of measures should be considered as risk factors and could be targeted to decrease the burden of CAS and stroke. Meanwhile, we should increase medical investment in rural areas to improve the prevention and treatment of diseases.

## Data Availability Statement

The original contributions presented in the study are included in the article/supplementary material, further inquiries can be directed to the corresponding author/s.

## Ethics Statement

The research protocol was approved by the Ethics Clerk Association of the First Hospital of Shanxi Medical University. Written informed consent was obtained from all subjects.

## Author Contributions

JZ and HS conceived and designed the study. XZ, YF, and JL performed the experiments. XN and WL acquired the data. XZ and TL did the analysis. JZ wrote the paper. HS and YF reviewed and edited the manuscript. All authors read and approved the manuscript.

## Conflict of Interest

The authors declare that the research was conducted in the absence of any commercial or financial relationships that could be construed as a potential conflict of interest.
